# Authors’ reply to: ‘preventing atrial fibrillation with SGLT2 inhibitors: time, sex, and substrate’ by Karakasis *et al*.

**DOI:** 10.1093/ehjcvp/pvaf072

**Published:** 2025-09-19

**Authors:** Damiano Fedele, Sara Amicone, Lisa Canton, Francesco Angeli, Matteo Armillotta, Luca Bergamaschi, Carmine Pizzi

**Affiliations:** Department of Medical and Surgical Sciences (DIMEC), Alma Mater Studiorum, University of Bologna, Via Zamboni, 33, Bologna 40126, Italy; Cardiovascular Division, Morgagni-Pierantoni University Hospital, Via Carlo Forlanini, 34, Forlì 47121, Italy; Department of Medical and Surgical Sciences (DIMEC), Alma Mater Studiorum, University of Bologna, Via Zamboni, 33, Bologna 40126, Italy; Cardiovascular Division, Morgagni-Pierantoni University Hospital, Via Carlo Forlanini, 34, Forlì 47121, Italy; Department of Medical and Surgical Sciences (DIMEC), Alma Mater Studiorum, University of Bologna, Via Zamboni, 33, Bologna 40126, Italy; Cardiovascular Division, Morgagni-Pierantoni University Hospital, Via Carlo Forlanini, 34, Forlì 47121, Italy; Department of Medical and Surgical Sciences (DIMEC), Alma Mater Studiorum, University of Bologna, Via Zamboni, 33, Bologna 40126, Italy; Cardiovascular Division, Morgagni-Pierantoni University Hospital, Via Carlo Forlanini, 34, Forlì 47121, Italy; Department of Medical and Surgical Sciences (DIMEC), Alma Mater Studiorum, University of Bologna, Via Zamboni, 33, Bologna 40126, Italy; Cardiovascular Division, Morgagni-Pierantoni University Hospital, Via Carlo Forlanini, 34, Forlì 47121, Italy; Department of Medical and Surgical Sciences (DIMEC), Alma Mater Studiorum, University of Bologna, Via Zamboni, 33, Bologna 40126, Italy; Cardiovascular Division, Morgagni-Pierantoni University Hospital, Via Carlo Forlanini, 34, Forlì 47121, Italy; Department of Medical and Surgical Sciences (DIMEC), Alma Mater Studiorum, University of Bologna, Via Zamboni, 33, Bologna 40126, Italy; Cardiovascular Division, Morgagni-Pierantoni University Hospital, Via Carlo Forlanini, 34, Forlì 47121, Italy

We are really grateful to Karakasis P, Antoniadis AP, and Fragakis N for showing interest in our meta-analysis on the role of sodium–glucose cotransporter 2 (SGLT2) inhibitors in preventing atrial fibrillation (AF).^1^ We confirmed a protective effect of SGLT2 inhibitors against AF (risk ratio (RR) = 0.86, 95% confidence intervals (CI) 0.77–0.96) but demonstrated that this effect may be attenuated in patients with heart failure with mildly reduced or preserved ejection fraction (HFmrEF/HFpEF) as compared with those with heart failure with reduced ejection fraction (HFrEF) or no heart failure at all (*P*-value for group difference = 0.01). In their Letter to the European Heart Journal—Cardiovascular Pharmacotherapy, the authors highlight several important points that may improve our understanding of the mechanisms by which SGLT2 inhibitors prevent AF.^2^ In particular, they suggested that sex, follow-up duration, and atrial substrate may also modulate the antiarrhythmic effect of SGLT2 inhibitors.

Regarding the influence of sex, they are right to highlight sex-specific risk factors for AF development and atrial cardiomyopathy progression. Indeed, the diverse hormonal milieu to which females are exposed throughout their lives also influences atrial remodelling and the functioning of ionic channels, as well as the different prevalence of thyroid diseases.^3,4^ Moreover, in females undergoing AF catheter ablation, more extensive low-voltage zones have been reported compared with males, indicating a more advanced atrial remodelling status.^5^ Hence, prompted by Karakasis and colleagues, we performed a meta-regression analysis on our data. The female prevalence across the 52 randomized controlled trials (RCTs) in our meta-analysis was 40% (95% CI: 0.38–0.43). Meta-regression analysis revealed a significant reduction in the protective effect of SGLT2 inhibitors against AF as female prevalence increased (β 0.023, 95% CI 0.008–0.038, *P*-value 0.002, residual I^2^ = 0%). The limitations of this non-prespecified analysis should be considered. Nevertheless, this finding supports the hypothesis that females may have a reduced susceptibility to AF preventive effect of SGLT2 inhibitors, possibly due to a more advanced atrial remodelling with a different path leading to HFpEF.^6^ Similarly, older age may also correlate with more pronounced atrial stiffness and fibrosis.^7^ Therefore, we completed our analysis with a meta-regression taking into account mean age in each RCT. The AF-protective effect of SGLT2 inhibitors diminished as the mean age in year of each RCT increased (β 0.044, 95% CI 0.015–0.072, *P*-value 0.002, residual I^2^ = 0%).

Moreover, Karakasis *et al*. also raised the question of whether the duration of follow-up may influence the efficacy of SGLT2 inhibitors, since this varied considerably across the included RCTs. However, a stratified subgroup analysis based on follow-up duration (≤3 months, 3–12 months, >12 months) showed no significant difference (*P*-value for group difference = 0.24). Similarly, a meta-regression analysis considering follow-up duration in weeks showed no significant interaction (*P*-value 0.567). Therefore, while there is relevant variability in follow-up duration across the included RCTs, it does not appear to have a significant impact on the results.

Finally, as emphasized by Karakasis *et al*. and reported among the limitations of our meta-analysis, it was impossible to differentiate between incident and recurrent AF since these data were not consistently reported in RCTs. Consequently, differences in the efficacy in primary or secondary prevention could not be directly analysed. However, our analysis showed that RCTs enrolling patients with a previous history of AF demonstrated reduced efficacy in preventing AF. This finding calls into question the ability of SGLT2 inhibitors to prevent AF in the secondary prevention setting, where atrial remodelling may already be too advanced to respond to upstream therapy.

In conclusion, females and older patients may receive a reduced benefit from SGLT2 inhibitors in the prevention of AF. This could be due to a severe adverse atrial remodelling, which is a central feature of HFpEF, more often encountered in females and elderly. Conversely, SGLT2 inhibitors seem to prevent AF independently of follow-up duration. We agree with Karakasis and colleagues that specific studies assessing the antiarrhythmic efficacy of SGLT2 inhibitors are needed, and these should include data on the degree of atrial remodelling and diastolic dysfunction. We particularly appreciated their suggestion regarding the importance of imaging markers of functional or structural atrial disease, such as strain or fibrosis. However, the accuracy of these techniques should be considered in relation to sex-specific variability,^8^ especially in light of the results of the meta-regression on sex.

## Data Availability

The data underlying this article will be shared on reasonable request to the corresponding author.
